# Human–Autonomy Teaming: Definitions, Debates, and Directions

**DOI:** 10.3389/fpsyg.2021.589585

**Published:** 2021-05-28

**Authors:** Joseph B. Lyons, Katia Sycara, Michael Lewis, August Capiola

**Affiliations:** ^1^Air Force Research Laboratory, Dayton, OH, United States; ^2^School of Computer Science, Carnegie Mellon University, Pittsburgh, PA, United States; ^3^School of Computing and Information, University of Pittsburgh, Pittsburgh, PA, United States

**Keywords:** human–autonomy team, autonomy, team, human factors, robotics

## Abstract

Researchers are beginning to transition from studying human–automation interaction to human–autonomy teaming. This distinction has been highlighted in recent literature, and theoretical reasons why the psychological experience of humans interacting with autonomy may vary and affect subsequent collaboration outcomes are beginning to emerge (de Visser et al., 2018; Wynne and Lyons, 2018). In this review, we do a deep dive into human–autonomy teams (HATs) by explaining the differences between automation and autonomy and by reviewing the domain of human–human teaming to make inferences for HATs. We examine the domain of human–human teaming to extrapolate a few core factors that could have relevance for HATs. Notably, these factors involve critical social elements within teams that are central (as argued in this review) for HATs. We conclude by highlighting some research gaps that researchers should strive toward answering, which will ultimately facilitate a more nuanced and complete understanding of HATs in a variety of real-world contexts.

## Background

Teams have long been valuable assets that help organizations address dynamic and complex objectives. In recent years, within the human factors, computer science, and robotics domains, the scientific community has sought to extend teaming concepts to human-machine partnerships. Advancements in computer science in recent decades have allowed researchers to consider the use of intelligent machines to fulfill viable roles within human teams (Sycara and Lewis, 2004). Synthetic agents are increasingly being used to augment and partner with humans in realistic tasks (Demir et al., 2017), which suggests that as a community we are on the cusp of breakthroughs in technology with an evolving philosophy regarding the nature of contemporary work and how it gets accomplished.

While considerable work has been done on human-human teams over the past several decades, the literature on human–autonomy teams (HATs) is less mature but has been growing in recent years. However, humans have accumulated more than a century of experience with machine automation which is now increasingly incorporating intelligence. Despite the great sophistication of many of these machines, an advanced autopilot, for example, our relations to them remain largely unchanged. While the pilot’s safety is dependent on the autopilot making sophisticated stability decisions under challenging conditions he is likely to treat it as part of the plane’s controls; setting heading and altitude much as one might select a floor in an automated elevator. The same pilot, however, would likely recoil upon being asked to take on a dangerous mission in which his trusted wingman would be replaced by an autonomous drone. In both situations, the pilot’s fate and safety are entrusted to a sophisticated decision making machine, yet for the autopilot this can be accepted without a qualm while the autonomous wingman arouses uncertainty and fear. We believe this distinction is qualitative and real and involves the degree of autonomy, evolutionary human predisposition toward cooperation (Bowles and Gintis, 2011; Tomasello et al., 2012), and distinct forms of neural processing associated with human cooperation (Mu et al., 2018; Xie et al., 2020). The autopilot performs a rigid predictable function which the pilot can easily verify while, in contrast, depending upon the autonomous wingman requires the pilot to cede control in the belief that the wingman will ‘have his back’ throughout the mission and raises concerns about the lack of a “human thinker” in the cockpit (Lyons et al., 2018). This raises issues beyond the mere technological aspects of the machine – will this machine help me when I need it? – do we have a common understanding of the threats? – can we communicate enough to address task dynamics? We argue that the two interactions involve separate modes of human behavior with reliance on the autopilot depending on normal forms of interaction while close cooperation (and eventual collaboration) with the wingman requires summoning social patterns of behavior evolved over our species’ history of group foraging (Tomasello et al., 2012) and warfare (Bowles and Gintis, 2011). In conventional interaction, a machine is treated as a tool but in highly interdependent interactions under proper conditions, it can come to assume the role of a teammate benefiting from the increased fluidity and coherence of joint actions made possible by human propensities for social cooperation.

The notion of humans and machines sharing authority to pursue common goals is a prominent research topic within the human factors and computer science communities (Cummings, 2014; Flemisch et al., 2019). This topic has acquired prominence in recent years because of its particular importance within the domain of autonomous car research (Li et al., 2016; Awad et al., 2018) and military research (Chen and Barnes, 2014). Sycara and Lewis (2004) have identified three general roles that machines can support within teams: namely, machines may support human teams (1) by supporting individuals as they complete their individual tasks, (2) by assuming the role of an equal team member, or (3) by supporting the team as a whole. The first of these roles has been researched to date in the context of decision support systems; however, the second and third are the subjects of increasing research since they exist at the heart of HATs. Recent work considers an agent as a replacement of a human within a human-human team (McNeese et al., 2018), essentially a synthetic human (agent). Other work has considered issues of team processes, such as dynamic trust building and adaptation in complex and dynamic environments (Nam et al., 2020). In terms of identifying the components of a machine teammate that humans may find convincing, relevant research (e.g., Wynne and Lyons, 2018) has considered the various psychological antecedents and outcomes of experiencing a technology as a teammate versus a tool. This last issue is crucial since it sheds light into the desired characteristics of a machine as a teammate and hinges upon differences between automation (tool) and autonomy (potential teammate). Thus, the current review examines HATs in one of three typologies: (1) as a means to reveal the psychological antecedents of HAT perceptions, (2) as a human teammate substitute and communicative team member, and (3) as an influence on the team dynamics of the team as a whole.

### Why Do We Need Human–Autonomy Teams?

The overall rationale for the use of HATs is that they may facilitate better performance relative to humans alone or machines alone, particularly under situations of high uncertainty (Cummings, 2014). A classic example is provided in Kasparov (2010), wherein novice chess players can team with an intelligent agent and outplay master chess players. Autonomous systems often have computational powers that outmatch human abilities in both breadth and speed, and they often have better sensors compared to humans (Arkin, 2009; Scharre, 2018) which can increase the speed of data processing and increase the breadth of such analyses. When autonomous systems are used in combination with humans in HATs they can serve as a force multiplier, for in theory, fewer soldiers will be needed to execute the mission (Arkin, 2009; Endsley, 2015; Scharre, 2018). HATs may be most necessary in open-ended missions where not all of the mission parameters can be specified *a priori* (Chen and Barnes, 2014). However, the potential benefits of HATs are not limited to the military domain.

Human–autonomy teams may increase psychological safety for contributing information, making guesses, brainstorming, or reporting uncomfortable information for humans. Psychological safety refers to “a shared belief that the team is safe for interpersonal risk taking … that the team will not embarrass, reject, or punish someone from speaking up” (Edmondson, 1999, p. 354). Autonomous systems don’t judge others, unlike humans. In fact, there is promising research exploring the use of robots in helping children with autism (Diehl et al., 2013) and supporting soldiers with PTSD via intelligent virtual agents (Kang and Gratch, 2010).

### Automation Versus Autonomy

The need for HAT arises from situations requiring tightly coupled interactions between humans and machines. To the extent the machines’ actions can be precisely predicted, a social context may not be needed to facilitate interaction, as for example, in stability augmentation of a control system. Where the conditions determining a partner’s actions are not evident, however, attributions involving capabilities, objective functions (goals), or other characteristics of the machine may be needed to establish a basis for cooperation. For example, in Chien et al. (2020), compliance doubled when participants were shown why a path planner wanted to re-route a UAV while at the same time an earlier correlation between trust and compliance was eliminated.

Because machines are deterministic, algorithmic entities, the distinction between automation and autonomy lies in the eye of the human beholder and is one of the key debates surrounding human-autonomy teaming. Automation represents technology that actively seeks data, transforms information, makes decisions, or controls processes in a narrow and well-defined task where the automation can be pre-programmed (Lee and See, 2004). In other words, automation is doing something in place of a human that the human no longer needs to do. Granted, the use of automation does not eliminate the need for a human, rather it changes the role of a human often to one of a supervisor of the automation (Parasuraman and Riley, 1997), which comes with its own set of potential challenges. In fact, decades of research has shown that the use of automation often adds considerable complexity for humans who must monitor the technology (see Parasuraman and Riley, 1997; Lee and See, 2004; Hoff and Bashir, 2015 for reviews).

A critical assumption of automation is that it does its job in the confines of what it was programmed to do, making it unusable outside of that particular context. Automation serves a specific role, and its use can be associated with increased performance and reduced workload in nominal conditions (Onnasch et al., 2014). The literature on automation has developed taxonomies for the types of behaviors automation can engage in (e.g., information acquisition, information analysis, decision and action selection, and action implementation; Parasuraman et al., 2000) as well as the level of human intervention that is acceptable for a particular automated response (i.e., levels of automation; Sheridan and Verplank, 1978). A particularly unique class of automation is adaptive automation which invokes an automated behavior based on a detected change in a human’s state (such as physiological changes indicative of high or low workload; Hancock et al., 2013; Chen and Barnes, 2014). In this case, an automated tool can be programmed with a target threshold and granted the authority to detect dynamic changes of the targeted variable along a continuum for which it will enact a preset automated response. Therefore, automation may be delegated higher or lower levels of task execution flexibility within a specific context, but it lacks the authority to decide which contexts and the conditions under which it operates as these are structured *a priori* by the designer or operator of the automation. Since automation performs repeatable behaviors in established domains, predictability of the automation is a critical factor influencing one’s trust of the technology (Lee and See, 2004; Hoff and Bashir, 2015). More specifically, automation ought to operate reliably via underlying algorithmic processes that are contextually appropriate and implemented as intended by the designer (Lee and See, 2004, p. 59). For tasks that are relatively simple, situation-invariant, repeatable, and low risk, automation can be highly beneficial. However, for tasks that are complex, context-dependent, fluid, and high risk, automation use may lose its benefits or lead to undesirable outcomes due to its inherent inability to handle situations outside of those for which it has been programmed.

Autonomy, in contrast, is capable of making decisions independent of human control (Vagia et al., 2016). Autonomy could be responsive to situations it was not designed for (i.e., autonomy is capable of learning and generalization) and it should possess some authority to direct its own actions, i.e., be goal-directed (Endsley, 2015). Autonomy focuses on decision making, adaptation to changing demands, and performance improvement over time with some level of self-governance (United States Air Force (USAF), 2013). Self-governance, adaptability, and learning are important features of autonomy relative to automation. A cogent definition of autonomy, placing teamwork at the center of autonomy, is provided by McNeese et al. (2018) who state that “autonomy is a technology that is capable of working with humans as teammates to include the essential task work and teamwork function of a human teammate” (pp. 1–2). Key features of the autonomy, in this case, involve taking on a role within a team and communicating with one’s teammates. Given the focus on teamwork and communication, HATs require intent information, shared mental models, and social affordances to enable communication and shared understanding. These same features are reflected in the interpersonal teaming literature.

### What Can We Learn From Human Teams?

In the past several decades, the research community has placed considerable emphasis on the topic of teams in organizations, and this has largely been driven by the fact that organizations are using teams to accomplish a variety of tasks. Teams are often formed to help organizations address the need for adaptability as teams are better at adaptation than the larger organizational units (Kozlowski and Bell, 2003). Similarly, HATs are envisioned to support greater adaptability to dynamic situations relative to human–automation interactions. There are many models that inform the science of teams. In the management science and organizational behavior literatures, teams are defined as: two or more individuals who perform organizationally relevant tasks, share at least one common goal, interact socially, have task interdependence, maintain and manage boundaries and roles, and are embedded in a larger organizational context that sets objectives, boundaries, constraints on the team, and influences exchanges with other teams (Kozlowski and Bell, 2003). Teams include features such as collective ambition, common goals, alignment of individual goals, high skill differentiation, open communication, safety, and mutual commitment (van der Hout et al., 2018). While teams operate as distinguishable (and measureable) work units partially independent of (but subsumed within) the organization, the organization constrains and sets the overall objectives of the team (Kozlowski and Bell, 2003). Similarly, HATs would acquire the overall strategic objectives of their parent organization (such as the broader military service within which the HAT resides, for example). Therefore, it may be inappropriate to expect that a HAT will evaluate goals broadly, but rather HATs should consider proximal goals within the context of the broader goal hierarchy of the organization. This constrains the space of inquiry to relevant task contexts of an organization and eliminates the need for discussing the concept of general artificial intelligence (AI). Yet, teams are subject to developing proximal environmental features and dynamics paving the way for team-level influences on individuals (Kozlowski and Klein, 2000). These may come in the form of normative influences, shared perceptions, and expectations of individuals that clearly have both top–down and bottom–up sequelae. These top–down and bottom–up dynamics are also likely within HATs.

Perhaps the best known model for the study of team dynamics is the *Input-Process-Output (IPO)* model (McGrath, 1964). Within this model, inputs (in the form of resources, constraints, skills, group composition, etc.) influence team processes (mechanisms that allow the members to combine their inputs) which mediate the relationship between inputs and outputs. Team outputs are a key element for any organization employing a team and they vary according to the organization’s goals/strategy. However, the team processes are what translate team inputs into outputs, and thus for the purposes of this review the emphasis is on the team processes that could be influential in the context of human–autonomy teaming. Team processes include coordination, cooperation, and communication (Tannenbaum et al., 1992), and these team processes can be used to measure and evaluate how teams process information, make decisions, and combine individual actions toward joint objectives (i.e., the process of team cognition; Salas and Fiore, 2004). Team processes are central to teams because they (1) influence the development and implementation of shared mental models, (2) shape individual motivation toward or away from team objectives (i.e., they shape intent), and (3) impact team cohesion (Kozlowski and Bell, 2003).

#### Team-Oriented Intent

Effective team members *share goals* with their team; thus, actions that signal this goal alignment (i.e., intent) are an important team process component. One of the risks associated with teamwork is that one team member could be less motivated toward team objectives than other team members and engage in behaviors such as social loafing. Showing motivation to pursue team goals (versus individual goals) should promote trust - the willingness to accept vulnerability based on the expectation of a positive outcome (Mayer et al., 1995) – within a team (Dirks, 1999). As trust from one team member toward another may have an influence on the degree to which trust is reciprocated (Serva et al., 2005), this greater trust should reduce social loafing and promote more motivation toward team goals. Further, intention-based information that suggests that a teammate is benevolent should increase trust of the teammate (Mayer et al., 1995; Serva et al., 2005).

Intent information can also come in the form of one’s desire to be part of a team. Team cohesion is a critical team process as it enables attraction toward the team and joint motivation on both a social and task level (Kozlowski and Bell, 2003). The link between cohesion and team performance is reciprocal – therefore, enhancements to team cohesion increase performance, which in turn, promote more cohesion (Kozlowski et al., 2015). The recognition of cohesion as a multidimensional construct dates back to the seminal work of Festinger (1950). He discussed cohesion as a culmination of factors, such as attraction to the members of a group, the activities of a group (task commitment), and the prestige of the group (group pride). Cohesion develops after the group has had an opportunity to work together or at least become acquainted with each other (Gosenpud, 1989; Matheson et al., 1996; Harrison et al., 1998). A meta-analysis (Beal et al., 2003) found that all three of Festinger’s (1950) original components of cohesion—interpersonal attraction, task commitment, and group pride—each bear significant independent relations to performance across many criterion categories. Whether human or machine, knowing the intent of one’s partner is an important team enabler.

#### Shared Mental Models

Shared mental models represent knowledge structures that may be shared among team members that allow team members to create accurate representations and predictions within a team task context (Cannon-Bowers et al., 1993). These representations can be oriented toward the equipment (i.e., material assets) within a team, the team task (procedures, constraints), the team members (skills, abilities, strengths, weaknesses), and the team interactions necessary within the team (roles, expectations, dependencies) (Cannon-Bowers et al., 1993). Shared mental models are important for team effectiveness because they facilitate a common framework from which to interpret the environment and the team’s progress in relation to shared goals (Cooke et al., 2013). Research has shown that teams perform better when they share mental models (Mathieu et al., 2000). Teams develop shared mental models through a process called team cognition, which is cognitive activity at the team level with the desired outcome of shared understanding among team members (Cooke et al., 2013). Communication between team members is the foundation for team cognition. Thus, communication is critical to understanding how well a team can recognize anomalies, adapt to them, and offer feedback within the team—all of which ought to impact team performance outcomes.

#### Communication

Communication is the cornerstone of teamwork as this is the mechanism through with teams translate individual action and effort into collection outcomes. Teams that communicate and monitor performance in relation to shared goals out-perform groups that lack shared goals and do not share intent toward those goals (Aubert and Kelsey, 2003). It is not sufficient for teams to share goals, but rather, the intent to support shared goals is a critical team process element that helps to direct individual contributions toward collective, team-level objectives. Communication herein is needed to convey this shared intent and to adjudicate performance monitoring. High performing teams tend to be more efficient in their use of questions, asking fewer questions yet still receiving all the necessary information (Urban et al., 1993). High performing teams also exhibit behaviors such as situation assessment and planning that help to achieve and maintain situation awareness (SA) (Orasanu, 1990). In other words, effective teams must collectively sense the environment, understand the environment in relation to shared goals, and act in accordance to those goals to support others on the team. One characteristic of effective teaming is to push information to one’s teammates before they need it (Demir et al., 2017), such that if the team is well-coordinated and communicates effectively there may be little reason to pull information from teammates. It has been observed that teams in which members provide unsolicited information to other team members generally perform better than those that do not (Urban et al., 1993; Johannesen et al., 1994).

In summary, team processes represent the gel that translates team inputs into collective outputs. Team processes allow for joint attention, shared goals and shared motivation toward those goals, and attraction toward the team members socially and in a task context. Effective team processes can: (1) signal shared intent toward collective goals, (2) promote team cognition in support of the development and maintenance of shared mental models, and (3) promote aiding and performance monitoring via communication. These same notions learned through the human–human teaming literature will likely apply to HATs as well.

### Defining Human–Autonomy Teams (HATs)

In the spirit of Nass et al. (1994) pioneering work that considered “providing the computer with characteristics associated with humans” an essential ingredient for eliciting social attributions, de Visser et al. (2018) consider ‘humanness’ (Haslam, 2006) to be independent of degree of autonomy. So, for example, a companion robot such as the baby seal Paro (Šabanović et al., 2013) might rank high on eliciting social responses while being devoid of autonomy. An automated cart in a warehouse, by contrast, might be highly autonomous in choosing goals and planning to optimize the placement of inventory while using collision avoidance to avoid humans, yet do so without any apparent social relationship. The challenges of human-autonomy teaming rest in developing (1) team-based affordances for fostering shared awareness and collective motivation, (2) an understanding of the types of tasks and interactions that stand to benefit from social cueing, and (3) developing techniques for using these cues to enhance HAT performance.

**FIGURE 1:**
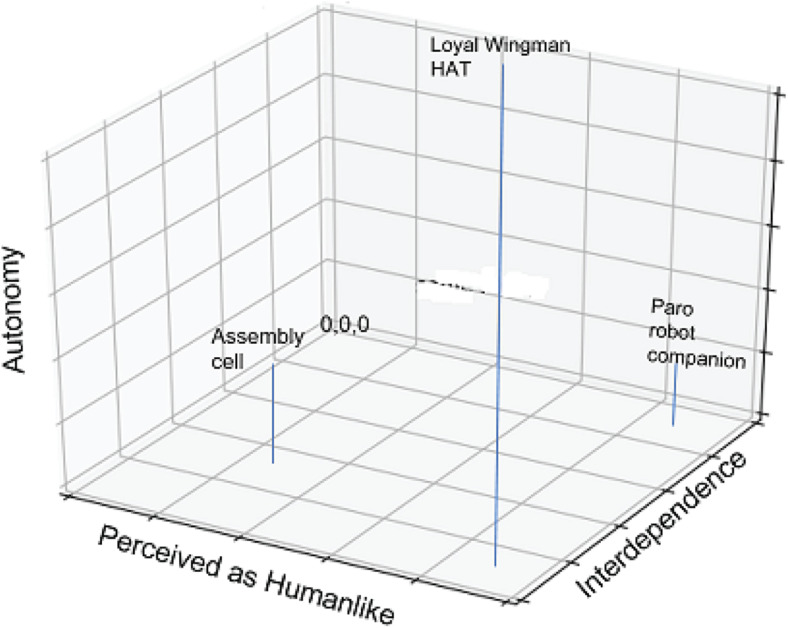
Human propensities for social cooperation are expected to facilitate human–machine performance where both machine autonomy and interdependence are high.

To answer the question of how human-machine interactions can be transformed to a HAT, we must first try to understand the characteristics of machine, task, and interactions that lead humans to consider and treat a machine as a teammate and the conditions under which this is expected to improve performance. Figure 1 shows a hypothesized relation between autonomy, interdependence, and perceived human-likeness. The companion robot Paros is high on perceived human-likeness but low on both autonomy and interdependence. The robots in a manufacturing cell are high on interdependence as they are continuously exchanging and positioning work pieces with the human and moderately high on autonomy. These interactions can be expected to benefit from adjustments and synchronization due to the human mirror neuron system (Pineda and Hecht, 2009) but should not require attributions of humanness. The Autonomous Wingman HAT exemplar, by contrast, is high on all three dimensions. Its degree of autonomy is so great that high interdependence requires an attribution of humanness and benevolence in order for cooperation to unfold without continual dysfunctional monitoring and second guessing by the human.

It is not yet clear what technology capabilities would be the ones that would make technology acceptable to humans, though research has begun to seek answers on this issue (see de Visser et al., 2018). Discovering these characteristics is important since robotics researchers can use these as the characteristics needed for modeling computational algorithms to make robots acceptable teammates to humans. This perspective, in considering the psychological view of machines as team members, is appropriate given the immense challenge of getting humans to accept machines as viable partners. “Today in almost all cases the limiting factor in human–agent interaction has become not computing cycles or connectivity (the machine side) but the user’s ability or willingness to communicate his or her desires and sift, organize, and interpret the machine’s response to satisfy them (the human side)” (Sycara and Lewis, 2004, p. 204). While this quote was made over 15 years ago, little process has been made to date to help inform researchers regarding what drives the beliefs of a machine as a teammate. However, given recent advancements in computational power, technology capability, and the ubiquity of robotic systems, it is imperative that the research community adopt common views of human-autonomy teaming and engage in integrative research to isolate and validate the components of effective HATs.

Researchers have long sought to understand the social interactions between people and machines. Teamwork is by definition social (in the sense that it involves more than one individual interacting with one another), as opposed to taskwork that can be performed by a single individual. In looking at human–autonomy teaming from a teamwork perspective, and asking what characteristics make a machine a good teammate, we assume that the technology will perform its assigned task correctly to an acceptable degree. Humans tend to respond to machines socially, even if not intended by the designer because humans apply social rules to machine interactions (Nass and Moon, 2000). For example, research looking at human-robot teams found that people prefer in-group robots over out-group humans (Fraune et al., 2017). From a psychological perspective these beliefs impact behavior, and thus must be considered in the context of HATs.

As a means to investigate the human teamwork factors composing HAT contexts, Wynne and Lyons (2018) developed a conceptual model of autonomous agent teammate-likeness (AAT) which detailed the factors that shape when a person perceives a technology as a “tool” (purely utilitarian beliefs [e.g., preprogrammed automation]) versus as a teammate. They define AAT as: “the extent to which a human operator perceives and identifies an autonomous, intelligent agent partner as a highly altruistic, benevolent, interdependent, emotive, communicative and synchronized agentic teammate, rather than simply an instrumental tool” (p. 355). In their model, the concepts of agency, benevolence, interdependence, communication richness, synchrony, and team focus are the key factors in shaping the perception of a technology as a teammate versus as a tool.

#### Agency

Agency, the capability and authority to act when and how the agent desires, is a necessary characteristic of a teammate. Humans consider agency to be an important part of what it means for a machine to be a teammate (Lyons et al., 2019). Agency is perhaps the most important distinguishing characteristic that differentiates automation from autonomy (as noted above) and consequently, it is one of the ingredients that computational agents must exhibit in order to enable humans to view them as teammates. In a survey of attitudes toward autonomous weapons, Arkin (2009) notes that a majority of surveyed humans believe that autonomous robots should have the ability to refuse orders that violate some rules of engagement – hence agency. In a review of human–agent teaming, Chen and Barnes (2014) state that agents should possess autonomy and be able to sense and act on their environment. It is difficult to be a teammate if one lacks the authority or capability to act when necessary, just as it is difficult to accept an agent as a true teammate if one perceives that the agent lacks volition.

#### Communication

In human teams, communication is one of the most important team processes since it facilitates formation of shared SA, shared mental models, and goal alignment (as noted above). The extent to which communication mechanisms support a wide range of emotional and social cues enhances team effectiveness (Hanumantharao and Grabowski, 2006) and can help facilitate the view of robots as teammates (Iqbal and Riek, 2017). HATs need to be able to establish common ground, communicate shared mental models, shared goals, and shared understanding of the world (Schaefer et al., 2017). HATs must be enabled to create the right levels of communication richness, appropriateness regarding communication content, and the proper timing. Research on etiquette has found that appropriate etiquette can enhance performance within human–automation interaction (Parasuraman and Miller, 2004). Some view this simply as the machine acting politely. In the context of HATs, however, machines will need to be much more than just polite, they will need to be appropriate – this appropriateness is the core of machine etiquette (Dorneich et al., 2012; Peters et al., 2019). Gratch et al. (2007) looked at what they labeled contingent rapport behaviors (i.e., rapport behaviors that are closely coupled to human actions) and found that contingent rapport behaviors were more important than the frequency of agent behavior in influencing human perceptions of the agent. Thus, behaviors of the machine need to be closely coupled to an appropriate human expectation in order to maximize their effectiveness. The technical challenge is to develop algorithms that will maintain coherence of the human actions and the communication utterances. The Natural Language Processing (NLP) community has made large strides toward this goal in dialogue based systems; however, in HATs there are also additional challenging aspects such as coherence of utterances with perceived actions (in other words, integration of computer vision and language) as well as issues of when is a good time to interrupt the human team mate which pose additional technical problems (Altmann and Trafton, 2002; Banerjee et al., 2018).

#### Shared Mental Models

Communication is used to create shared metal models in a team. Human teams that share mental models are better equipped for common interpretations of dynamic contexts (Cannon-Bowers et al., 1993; Salas and Fiore, 2004; Cooke et al., 2013). In other words, they are in synch with the situation and what actions must be taken in a given situation. Having these capabilities will allow the HAT to begin to anticipate the needs, actions, and future problems within the team. Anticipation of needs and future actions is a critical teaming function (Liu et al., 2016; Demir et al., 2017; Iqbal and Riek, 2017; Chen et al., 2018) since it allows mutual adaptation and a greater degree of team coordination and synchronization (this has also been labeled fluency; Hoffman and Breazeal, 2007). Shared mental models is more than merely all team members sharing every bit of information with one another; such a strategy would be highly inefficient both in terms of computation and communication bandwidth. Moreover, such a model amounts to full centralization of the knowledge of the team, which obviates the need to have multiple individual teammates. In contrast, Lewis and Sycara (1993) made an initial attempt to create a computational shared mental model among specialists that would be computationally efficient. An effective machine partner will need to understand what information is important to share with a human partner and what the most effective timing for sharing the information is, the latter being just as important as the former from a team dynamics perspective.

#### Intent

While shared mental models reflect the knowledge of the team regarding its environment, goals, and plans, shared intent reflects the particular sub-goal and action that a teammate may undertake in the near future. Communicating intent to support shared goals is a necessary process in human teams and this importance could extend to HATs. Initial research pointing in this direction has examined communication content in human teams in time critical and dynamic environments such as aircraft crews (Orasanu, 1990) and foraging scenarios (Sukthankar et al., 2009). Research on these teams has found that the highest performing teams are proficient at conveying high-level intent among the team members versus sharing all possible information (Sukthankar et al., 2009). This decreases information overload and allows the teams to focus on the important issues that must be addressed.

Lyons (2013) states that machines (i.e., robots) should communicate their intent in relation to humans who interact with them. This information should include information about shared goals. Notably, information regarding intent could involve planned actions for a teammate to accomplish her own goals, or a teammate’s goals, if the teammate is perceived as being incapable of accomplishing them herself (intent expressing helping behaviors). Task-based intent could present communications that a partner is pursuing a particular course of action (e.g., “I plan to complete the tasks in area 3”). Intent in this sense is similar to the highest level of transparency in the Chen et al. (2018) Situation-Awareness-based Transparency (SAT) Model as it provides a projection of future behavior. Studies have shown, for instance, that failure to signal intent can cause delays in activities such as hand-overs between humans and robots (Cakmak et al., 2011). Work in computer science and robotics on conveying intent has mainly focused on plan recognition (Sukthankar and Sycara, 2006, 2008) and Inverse Reinforcement Learning (IRL), where an agent views human decision trajectories, and under the assumption that the human obeys a Markov Decision Process (MDP), infers the reward function of the human (Hughes et al., 2020). Under an MDP, the reward function expresses the reasoner’s intent, since she will follow a course of action to maximize the reward. However, most of the IRL literature assumes (a) that the trajectories are given to the agent *ex post* facto for a static computation of the reward function, (b) that the agent uses the inferred reward function so as to learn to imitate the behavior of the human, and (c) that the preferences/reward of the human is static. However, these assumptions do not hold in dynamic HAT missions. In recent work (Hughes et al., 2020), researchers have demonstrated an algorithm that allows an agent to infer in real time changing human preferences (rewards) as the environment changes dynamically. This is the first system to do so, and it opens the way to develop agents that, knowing a human’s intent, can reason about the best way to plan their own actions to enable the team to address a changing and uncertain environment.

Advances in AI over the past 20 years have come largely from the adoption of these Markov decision models. More recently, Deep Neural Networks (DNNs) have been incorporated as function approximators (Deep Reinforcement Learning, or DRL) allowing the solution of even larger problems. This advance has enabled difficult to believe feats, such as programs that acquire human to superhuman skill at Atari games (Mnih et al., 2015) just by playing them, choosing actions based on the pixels on the screen. It is these highly capable AI programs that offer the most promise for dealing with the complexity and non-linearity of real time military and civilian systems.

Unfortunately, DRL is inherently opaque and difficult for humans to predict or understand. We find ourselves compelled to attribute causation even when it is clearly absent (Thines, 1991). The result is that our most capable AI teammates may also be the most difficult to understand and work with. One common result (Iyer et al., 2018; Anderson et al., 2019) is that while some DRL agent actions are easy to understand and predict, others seem completely alien and counter to our expectations. This perceived “strangeness” has led to a taxonomy (Chakraborti et al., 2019) of explicability-consistency with believed intent, predictability-consistency with predicted action, and legibility-distinguishing between alternate intentions. For example, a PacMan that neglected nearby edible pellets to move closer to a hostile ghost (Iyer et al., 2018) would appear inexplicable and its behavior unpredictable. Other peculiarities of learned behavior such as spacecraft in Team Space Fortress (described in the Sample HAT Research section) that learn to spin about as they fly may not preclude teamwork. The spinning, possibly an artifact of limiting collision detection to the center of the ship, is inexplicable but does not impede cooperation with a human shooter. While research in human–autonomy teaming typically poses the question of how autonomy can adapt to, promote, or better manage interaction with humans, the characteristics of high performing DRL agents are such that the burden of adaptation may need to be borne from both sides if the partnership is to work. This problem of promoting trust and teamwork with an inherently opaque teammate is one that HAT research must confront to take advantage of our most capable potential partners.

Communicating intent is one way to reduce opacity of agents. This can be expressed by communication that reflects expressions of support, benevolent intent, and adherence to goals that are a priority for the partner (e.g., “I can see you are struggling with this task so I will help you” or “I plan to complete the tasks in area 3 because I know they are important to you”). Expressions of benevolence, the belief that an entity has one’s best interest in mind and plans to act on those intentions, is a fundamental aspect of the human trust process (Mayer et al., 1995). Similarly, Lyons et al. (2021) found that providing participants with information regarding the stated social intent of the robot influences trustworthiness perceptions of the robot. Stated social intent characterized by self-sacrifice (perhaps the quintessential form of benevolence) was related to higher benevolence and integrity beliefs relative to other types of stated social intent (Lyons et al., 2021). The intent-based programming of machines matters. However, Lee and See (2004) noted that because automation has no intentionality or agency, ascriptions of automation benevolence (or purpose) are really ascriptions toward the automation’s designer.

As technology transitions from automation to autonomy emulating more human-like characteristics, humans may begin to ascribe intentionality toward the autonomy in a genuine way. There is currently little research on algorithms that would recognize shortcomings of human skill or errors in human performance that would allow the robot to intervene and express its willingness and intention to help. Such work is needed to test (a) the ability of the robots to diagnose human task failures, (b) the ability of the robot to perform the correct helping behavior, and (c) the extent to which such behavior makes the human consider the robot as a teammate.

#### Interdependence

Interdependence is a core aspect of teamwork (Kozlowski and Bell, 2003). When working toward shared goals, team members must have common task work and interdependence on each other for team outputs. Interdependence is also a key need within human–machine partnerships (Johnson and Vera, 2019). Working interdependently means that humans and machines will undertake roles and divide teamwork in interdependent tasks that each one must perform. Similarly, in operations research and computer science there are multiple works in task allocation in agent-only teams (Luo et al., 2011), but there is a dearth of literature in task allocation in multi-individual human-autonomy teams in complex environments. Besides task allocation, interdependence implies shared responsibility for task accomplishment. In its extreme, shared responsibility is shared control where a human and an agent, for example, push a box together (Wang and Lewis, 2007), or where a passenger switches control of a driverless car with the car controller (Stanton and Young, 2005). In the box pushing example, synchronization of movement of the robot and human is very challenging, and in the car example, the challenge mainly lies in when to switch and how to safely and robustly make the handoff.

In summary, a HAT requires five essential elements. First, the machine must have a high level of agency to act as a teammate. Second, the machine must be communicative. Humans are assumed to also be both agentic and able to communicate. This communication should [third] convey information that allows the human teammate understand the intent of the machine. Fourth, the human and machine should share mental models of the team assets, team strengths/weaknesses, division of labor, and task context. Finally, a HAT is characterized by interdependence between the human and machine. The section that follows will discuss some of the extant literature regarding HATs. Rather than organize this section according to the HAT elements discussed above, this section was organized based on the taxonomy mention at the outset of the review, namely psychological antecedents, teammate substitutes, and factors that influence the team as a whole.

### Samples of HAT Research

The following section is not intended to be exhaustive of the HAT literature, but rather it examines some of the recent HAT studies and programs to aid in our understanding of the extant literature. These examples adopt the HAT typologies mentioned in the early sections of the manuscript: (1) as a means to reveal the psychological antecedents of HAT perceptions, (2) as a human teammate substitute and communicative team member, and (3) as an influence on the dynamics of the team as a whole.

#### Psychological Antecedents of HAT Perceptions

Taking the psychological perspective of HATs, several studies have examined the AATs dimensions to develop valid and reliable scales to assess these constructs. Lyons et al. (2019) first conducted a qualitative study to ask workers about their experiences in working with technologies they considered “intelligent.” When asked about whether or not these technologies were teammates versus tools, about one third responded with the belief that the technology was a teammate. The authors further asked several open-ended questions regarding why the participants thought the technologies were teammates, or alternatively, if the participants believed their technology to be a tool what it would take for the technology to be considered a teammate. These open-ended questions were then coded using qualitative analysis techniques by four independent coders who sought to organize the results into themes. The six dimensions of the AAT model played out prominently in the data. In addition, the broad concept of humanness was also a dominant theme within the data, but it was not clear if the participants believed some of the AAT dimensions to be examples of humanness (see Lyons et al., 2019). This initial study suggested that the AAT model proposed by Wynne and Lyons (2018) had some validity.

This initial study was followed up by a second study to examine the psychometric properties of initial AATs scales (Wynne and Lyons, 2019). The psychometric study resulted in scales to index perceptions of the AAT dimensions. These scales were, in turn, used in a third study wherein they were incorporated into a narrative research design. Lyons and Wynne (2021) created narratives of technology that were high and low on the AATs factors. Participants reviewed the narratives and measured participants’ perceived AATs dimensions, teaming perceptions, and commitment to the technology. Results from the study demonstrated that the AATs scales were sensitive to the narrative manipulation. Further, the AAT dimensions of interdependency, communication richness, and team focus were uniquely related to higher commitment to the technology. While these initial AAT studies are promising, additional research is needed to fully examine the AAT model. However, these findings provide broad support the importance of agency, teammate-oriented intent, interdependence, shared mental models, and communication.

#### Human Teammate Substitute

Taking the perspective of an agent replacing a human role in a team of humans, research by Demir et al. (2017) examined the role of a synthetic teammate in team process and performance using an unmanned aerial vehicle simulation task. They used a control condition consisting of novice human subjects, an expert team consisting of an expert confederate playing as one of the three roles (a pilot, navigator, and sensor operator), and a synthetic condition wherein one of the team members was a synthetic, ACT-R (Anderson and Lebiere, 1998) based agent. They found that the synthetic-agent condition performed the same as the control condition – which is useful to demonstrate that a partially synthetic team can perform at the same level as an all-human (though novice) team. Both the synthetic and the control conditions performed worse than the expert team, which is understandable. One really interesting finding, however, was that the synthetic teams tended to push less information. This is critical because effective teams push more information than they pull, signaling an ability to anticipate one’s needs in this experimental paradigm. Using the same research paradigm as above, McNeese et al. (2018) found that synthetic teammates were slower to respond to changes in the experiment and they tended to process targets slower than all-human teams. Taken together, the results of these studies show that teams comprising autonomous teammates may have challenges regarding communication and experience potential bottlenecks when unforeseen events take place, suggesting the need for shared mental models.

**FIGURE 2:**
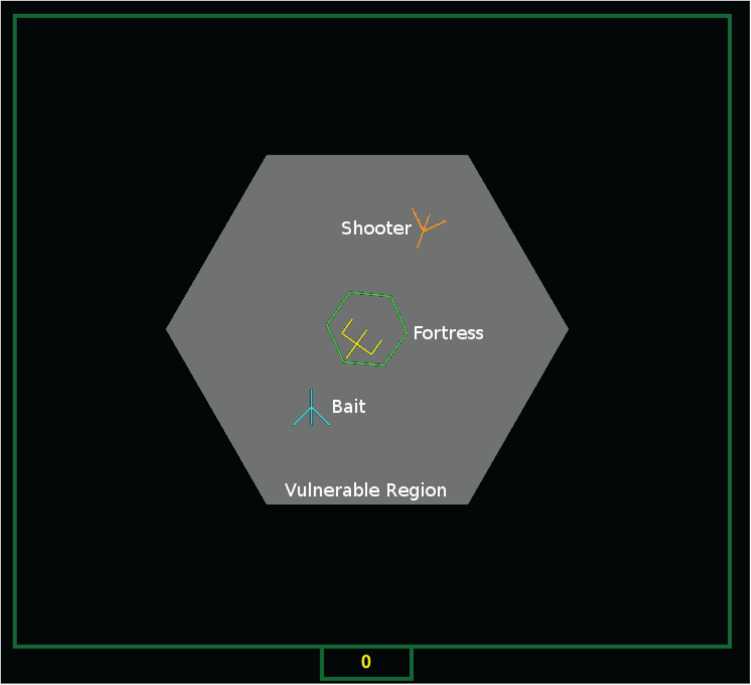
Team Space Fortress interface. The Fortress has dropped its shield to shoot at the Bait while the Shooter has begun firing at the vulnerable Fortress.

In recent work, Li et al. (2020) examined teammate collaboration and action adaptation during the mission in tightly coupled, time stressed teamwork. In collaborative tasks, the final outcome is determined by individual level (e.g., skill level, motivation, and personality) and team level (e.g., communication, team coherence, and complementary strategy) criterion of interest. It is also worth noting that in high quality teams the adaptation is mutual among team members (Salas and Fiore, 2004). Therefore, the problem of measuring the process of mutual adaptation within teams is very challenging because adaptation is non-stationary. Identifying teammate contributions is complicated by differences in play due to adaptation to other teammates leading to equilibria in which each player’s actions and contributions may be quite distinct from team to team. In this work (Li et al., 2020), the researchers identified the components of team adaptation in more detail, with respect to not only the final outcome (performance measure) but also the adaptive behaviors that occur as the mission evolves, a quantification that is novel in the human factors and multi-agent literature. Space Fortress (Agarwal et al., 2019), a game that has been used extensively for psychological research, was adapted for investigate teams. Team/Co-Op Space Fortress (TSF) is a 2-D cooperative game where two players control spaceships to destroy a fortress. An example interface is given as Figure 2. The fortress is located at the center of the screen while two airships are controlled by human players via XBox controllers (or can be autonomous agents). The first airship, the *bait* entering the hexagon area, is locked on and shot at by the fortress. The fortress becomes vulnerable (its back shield opens) while it is firing. The other teammate in the role of *shooter* can now shoot at the fortress to try to destroy it. The team performance is measured by the number of fortress kills and the task is highly interdependent as one team member must assume a more vulnerable role in order for the other to fire on the fortress.

Results showed that human players who adapted their policy when switching partners also led to better team performance. This finding indicates that individual adaptive actions during the course of the mission contribute to team coordination and therefore improve team performance. These findings suggest that an effective agent in a human-agent team must both learn to adapt to teammates and to adapt in such a way that their actions complement the teammate’s behavior. Researchers have developed agent learning strategies via Deep Reinforcement Learning. Agents learn to play with other agent teammates in TSF and make similarity judgments of the play trajectories of agent teammates, categorizing them into player types. When faced with an unknown human teammate, the agent recognizes the human type and adapts its strategy appropriately, based on similarity judgments and experiences of playing with previous agent teammates (Sycara et al., 2020). A challenge of adaptation on the part of the human is that, as agents train via self-play or all-agent team play, they come up with new strategies, some of which are unintuitive to humans, and therefore humans must adapt to them quickly.

Other research has examined autonomous agent collaboration within a multi-agent system, for example the RETSINA system (Sycara et al., 2003). To have an agent that is capable of both monitoring its own performance/goals and the performance/goals of other agents (including human agents), the agent must have a model of the performance or goal and a mechanism for monitoring it. Methods such as the RETSINA multi-agent framework have been used in DoD planning to aid humans in evaluating a humanitarian crisis, supporting plans for evacuation, monitoring activity, and dynamically re-planning (Sycara and Lewis, 2004).

#### Influence on the Team as a Whole

Within the Air Force, researchers have developed single operator control strategies of multiple unmanned aircraft systems using a human-autonomy team design. Using the Intelligent Multi-unmanned vehicle Planner with Adaptive Collaborative/control Technologies (IMPACT) platform, Draper et al. (2017) enabled a human operator to juxtapose higher-level intent with intelligent agents to manage Unmanned Vehicles (UxVs) in an operational mission context. The IMPACT system works through a combination of intuitive human-machine interface features, a play-calling framework that allows users to represent higher-level mission intent, and intelligent agents that can translate the higher-level intent into task execution for the UxVs. The agents leverage formal domain models (to represent a task context) and multi-objective optimization algorithms when aligning options for plays that are visualized and actioned by a human operator (Hansen et al., 2016). Under nominal conditions the human operator can orchestrate the mission space and convey mission intent to the system and allow the agents to optimize responses; notably, the human can modify any aspect of a play at any time, and the system will autonomously modify the ongoing play by dynamically re-allocating assets where appropriate and maintaining the human’s intent. Furthermore, the level of decision authority of the agents can be accelerated to allow the agent to automatically respond to agreed upon stimuli (Draper et al., 2017). The automatic response to *a priori* events would be considered automation using the above constraints; however, the automatic and dynamic response of the agents to a novel demand in ways that are not known *a priori* would classify it as autonomy. In this research the agent has a high level of agency, communicates changes and awareness of the situation, and shared intent is designed into the system in advance (in the form of human-accepted scenarios in which the agent can autonomously execute plays).

Also within the Air Force, research has been done to examine interaction methods between pilots and autonomous wingmen (robotic aircraft flying alongside a manned fighter platform). A study by Panganiban et al. (2019) examined the style of communication from the robot aircraft using natural language. They found that communication styles from the robot that emphasized benevolent intentions were able to reduce pilot workload and improved perceptions of the team collaboration using a sample of students, showing the benefits of rich communication and team-oriented intent. Further, the Autonomy for Air Combat Missions (ATACM) program developed a tactical battle manager (TBM) to enable a pilot to command multiple unmanned aircraft from a cockpit in contested domains (Schumacher et al., 2017). The ATACM technologies enable a pilot to maintain operational control of a team of unmanned aircraft in a tactical situation through a system of human–machine interfaces, autonomous aircraft behaviors, and AI-based multi-agent controller showing interdependence and communication.

Considerable work has been done on human–robot interaction in proximate space. Iqbal and Riek (2017) discuss methods to promote awareness of the action of others (e.g., a human working with a robot) in proximate space. Specifically, they have developed methods to allow the robot to anticipate and react to complementary actions from a human partner and these methods have been shown to improve human-robot coordination and performance in tasks such as hand-overs and joint work in small, shared spaces. These methods were also applied to understanding team communication dynamics and were found to result in greater human–robot team synchronization relative to interactions without the algorithms (Iqbal et al., 2016). The benefits of human-aware algorithms have also been demonstrated by Lasota and Shah (2015) and Liu et al. (2016). Thus, it appears that awareness of one’s teammate is an important feature in HATs, as noted above. Also, notable research is being lead to examine the role of robotic teammates on inter- and intra-group perceptions and dynamics (see Fraune et al., 2017).

In summary, some empirical studies have been conducted in the context of HATs and these tend to focus on psychological factors that shape HAT perceptions, substituting machines in team roles which were previously occupied by humans, and research to examine how HATs influence the team as a whole. These studies show the importance of agency, team-oriented intent, shared mental models, communication, and interdependence within HATs. However, research in the HAT is at its infancy and considerable research is needed to further our understanding of HATs.

### Research Gaps

We have identified the features of human–autonomy teaming and why HATs could add value to contemporary operations; yet, HATs are an emergent research topic and several gaps remain for the research community to fill. The following section will discuss some of the notable research gaps within this space.

One challenge in human–autonomy teaming is the capability of the agent to communicate its intent (Schaefer et al., 2017). As noted above, communicating intent could be task-focused to represent the projection of activity and focus of the machine’s attention, or it could signal the machine’s intention on providing help to the human. In addition, the agent must be able to infer the human’s intent so as to adapt its own goals and actions toward joint team goal fulfillment. No one debates the importance of communicating intent, but understanding how to do this effectively and what impact it has on HAT performance is a critical need within the research community. This is a gap that will be filled with theoretical and experimental research, both in the development of computational models for agents and experiments involving HATs. As mentioned earlier, Lyons (2013) states that machines should and could (Lyons et al., 2021) communicate their intent and goals with their teammates to affect human teammate perceptions, and such findings provide a first step in instantiating intent in a seemingly autonomous teammate and exploring how this apparent intent affects HAT interactions. Research has begun to investigate HATs which comprise intelligent agents that emulate critical task-based attributes of an effective human teammate (McNeese et al., 2018), and future work ought to investigate how autonomy intentionality can be manipulated and investigated in the lab to isolate the mechanisms responsible for intentionality perceptions and their sequelae.

Second, how can researchers enable team-level flow, peak performance, and positive psychological experiences such as cohesion within HATs? Team flow is an emergent topic within team sciences and requires volitional attention and action toward team goals/activities (van der Hout et al., 2018). How can we establish and maintain unit cohesion with the introduction of machines as teammates? A good beginning is to start operationalization of the components of cohesion, namely interpersonal attraction, task commitment, and group pride so as to be able to develop computational models of cohesion and HAT experiments. But is there a possibility that introduction of robots in a human team may disrupt team cohesion?

Third, mutual monitoring is a feature of effective teamwork. However, research shows that agents are trusted less than humans when directly compared to humans in the same study (Johnson and Mislin, 2011), so identifying how the introduction of an agent influences the broader team is a critical endeavor. If an expectation is violated by a machine partner, how does the machine repair the trust they may have had with the human partner (de Visser et al., 2018)? Liu et al. (2019a, b) have started answering this question via an algorithm whereby the system (a multi-robot swarm) repairs itself upon losing human trust. Another way is for the system to explain its behavior (become more transparent), since inability to understand system behavior may have been the reason for the human loss of trust. There is budding research in the computational community, especially involving neural networks that are opaque in enabling the system to explain its behavior (Annasamy and Sycara, 2019).

Fourth, given the criticality of team process as a determinant of HAT effectiveness, methods are needed to facilitate joint attention between the humans and machines. Novel interfaces are needed to convey where the machine is focusing its attention, particularly for distributed interactions (Iqbal and Riek, 2017). Methods to signal joint attention could be instrumental in ensuring alignment of mental models as the HAT experiences novel stimuli. Computer Vision algorithms output bounding boxes and also points, e.g., outlining lips that are recognized as smiling (De la Torre and Cohn, 2011), could help in this regard. Novel research needs to be done to test the effectiveness of these techniques in conveying joint attention in HATs and to examine their impact of shared mental models.

Fifth, methods are needed to facilitate joint understanding of states like confusion, mutual agreement, concern, and even emotion between humans and machines. Such socio-emotional cues are critical features within teams as they are used to determine and evaluate common ground, problems, and conflict among team members (Hanumantharao and Grabowski, 2006). Neurophysiological measurements such as EEG and fNIRS have been recently used along with appropriate computational models of the output signals to construct indicators of workload, focus of attention, and emotions (McKendrick et al., 2017). Further, advanced communication methods could shape shared awareness of these states in HATs.

Sixth, how do we establish shared accountability within the context of a HAT? There are often asymmetries that exist when comparing expectations and consequences of machine-based actions/decisions versus human ones. Research shows that robots for instance, are given less blame when an error occurs relative to human operators. This is supported by recent survey work which found that people tend to ascribe less blame to an autonomous car (relative to a human driver) when both parties are at fault for an accident (Li et al., 2016). However, the blame on robots increases as the robot’s level of autonomy increases (Arkin, 2009).

Seventh, what is the optimal level and method of training to team with machines so that an effective combination of skills within the team can be achieved? Similar to human teammates, it is a highly flawed belief that HATs will initially work without error. In contrast, researchers and practitioners need to consider what experiential knowledge is necessary to equip humans with awareness of the machine’s capabilities and limitations in various contexts (Christensen and Lyons, 2017). This will require a degree of self-awareness and transparency on the part of the machine, so it can be aware of its capabilities and limitations and make them known to humans. Initial work on enabling machines to communicate uncertainty on their decisions, explain their decisions, and make human partners aware of their capabilities via learning is beginning to appear in the computer science and robotics literature (Chien et al., 2012; Phillips et al., 2016).

## Conclusion

As technologies evolve from assistive tools to collaborators, researchers are beginning to reorient their focus from human–automation interaction to human–autonomy teaming (de Visser et al., 2018). In this review, we pursued four challenges to extend the HAT literature. We began by deciphering the differences between automation and autonomy and then segued into a brief review of linkages between human–human teams and HATs. We then defined HATs followed by a summary of recent work on HATs which classified studies based on the psychological antecedents of HAT perceptions, studies on using intelligent machines as substitutes for human teammates, and research on the HAT’s influence on the team as a whole. We then concluded by offering research gaps that scientists ought to fill.

It is our hope that researchers will use this review as a springboard for identifying where the state of human–autonomy research has come from, where it resides (as well as the burgeoning technologies that can be leveraged in research), and what questions need answering for a more thorough understanding of human-autonomy teaming. Readers should take away the following key points as they strive toward HAT research. Machines used as part of HAT must have some decision authority (beyond automation) to determine action appropriately. The task scenarios that seek to invoke HATs must emphasize interdependence between the human and their machine partner(s). Yet, while both agency and interdependence are necessary conditions for HATs they are not sufficient. HATs are characterized by team-oriented intent signaling, shared mental models, and rich communication affordances which, no doubt, form the underlying basis for the development and management of team-oriented intentions and shared mental models. HATs are no longer limited to science fiction movies, it is time for the research community to dive into this interesting, scary, and provocative topic.

## Scoping Statement

The current manuscript sought to review the concept of Human–Autonomy Teaming (HAT), a burgeoning topic within human factors, robotics, and computer science. Like any review, this is an ambitious undertaking and, as such, the authors offer a statement to appropriately scope the manuscript and to calibrate the reader. First, this manuscript does not pursue the issue of generalized artificial intelligence. Second, as a major user of tightly coupled human–machine systems, the US military has been a primary sponsor and promoter of HATs. Because the majority of work in this field to date has come from military research, this is where we draw most (but not all) examples and applications. Thus, one must use caution when extrapolating lessons learned from this review beyond a military domain. Third, there are many team frameworks and typologies within management science, but for the purposes of this review, we focus on the internal dynamics of teams as these dynamics will have the greatest relevance for HATs. With this emphasis on team dynamics we concentrate on the influences that shape team member *intent*, *shared mental models*, and *communication*. In doing so we acknowledge that factors such as team size, team leadership, team typology, top–down team goals, and demographic makeup of team members all shape team functioning. We assume that within military HATs: (1) there will be higher-level objectives (independent of the task objectives) that constrain the HAT goals, (2) team leadership will mostly reside with the human though it may be somewhat fluid depending on the situation and the relative capabilities of both the human and the machine, (3) the HAT will be used in a task context which is constrained by higher-level mission objectives, and (4) the HAT will comprise at least two types of individual entities (human and machine) though it is not limited to only two entities. This review does adopt the view that teams pass through different stages along a lifecycle of a team (Tuckman, 1965), and thus the lifecycle of a HAT is a necessary and relevant consideration.

Given the above, the manuscript sought to pursue a number of HAT-oriented challenges. One of the central challenges in HAT research is identifying the characteristics of machines and tasks needed to elicit human treatment of a machine as a teammate. A central tenet of this review is that machine-based intent, shared mental models, and social affordances for communication and understanding are necessary for HAT development and maintenance. A second challenge in HAT research is in distinguishing automation from autonomy – this is critical because autonomy forms the basis of a HAT. A third major challenge for HAT research is in understanding what features of human–human teaming can be leveraged and extended for HATs. A fourth challenge involves defining the essential features of a HAT. A final challenge involves communicating research needs in the context of HATs for the research community. The current manuscript sought to the address these challenges while discussing a few examples of HAT research and technologies to demonstrate the state of the art in this emerging domain.

## Author Contributions

All authors listed have made a substantial, direct and intellectual contribution to the work, and approved it for publication.

## Conflict of Interest

The authors declare that the research was conducted in the absence of any commercial or financial relationships that could be construed as a potential conflict of interest.
